# Encoder-free hexapod dead reckoning on sand using learned inertial correction

**DOI:** 10.3389/frobt.2026.1920986

**Published:** 2026-08-26

**Authors:** Mingyu Pan, Maxwell A. Rollins, Samuel H. Yang, Kathryn A. Daltorio

**Affiliations:** Department of Mechanical and Aerospace Engineering, Case Western Reserve University, Cleveland, OH, United States

**Keywords:** inertial measurement units, legged robot, localization, machine learning, proprioceptive sensing

## Abstract

For inexpensive legged robots traversing remote natural terrain, minimal-sensing localization strongly affects autonomous range and search efficiency. Although proprioceptive sensors such as inertial measurement units (IMU) are compact and widely used on robotic platforms, direct integration of noisy inertial measurements causes small sensor errors to propagate into large pose estimation errors, limiting their use as independent dead reckoning sensors. In this work, we address drift in encoder-free dead reckoning by combining body-frame acceleration profiles from a compact multi-IMU array with commanded motion parameters in a convolutional neural network (CNN) bias estimator. The estimator predicts the difference between commanded and realized platform displacement, without using joint encoders or explicit terrain labels. In outdoor local search experiments with an encoder-free hexapod robot, the proposed approach reduces mean dead reckoning drift by approximately 41% on grass and 59% on sand relative to an idealized commanded-kinematics baseline. These results indicate that a platform-specific dataset collected in controlled indoor terrains improves outdoor dead reckoning performance, even when the deployment terrain is not fully represented in the training dataset. This method provides a low-cost, compact proprioceptive drift reduction method for minimally instrumented legged robots when exteroceptive localization is unavailable due to cost or environmental conditions. We expect this approach to be helpful in developing future low-cost robots for scrambling across extraterrestrial, underwater, or underground granular media where leg slip is both common and hard to predict.

## 1 Introduction

Localization is a central challenge for small, low-cost legged robots operating in remote or unexplored natural terrain. Accurate position estimation enables systematic area coverage (Chin et al., 2016), efficient local search (Frosi et al., 2023), and self-docking for recharging (Page et al., 2021). However, many conventional localization methods rely on extensive sensory payloads such as cameras or Global Positioning System (GPS) that increase the platform’s size, cost, and environmental dependence. In contrast, small legged animals forage (Lima Vieira et al., 2024) and explore (Pearce-Duvet et al., 2011) effectively with much smaller body scales, suggesting the advantage of compact sensing methods for small robotic platforms. Terrains where legged locomotion is more useful also degrade simple dead reckoning methods. Vegetation, uneven terrain, and granular media introduce sinkage, slip, and substrate-dependent motion errors, causing the realized body displacement to differ from the commanded displacement. As a result, using commanded motion alone provides inaccurate position estimates, while directly integrating inertial measurements causes unbounded drift accumulation. In this work, acceleration profiles from low-cost body-mounted IMUs are used as motion signatures for a CNN-based bias estimator. We evaluate whether this approach reduces dead reckoning drift on a legged robot traversing two kinds of natural terrain: sand and grass.

Proprioceptive sensors have the advantage of being low-cost, compact, high bandwidth, and less dependent on external conditions (e.g., lighting, weather) than exteroceptive sensors, making them common in robotics. By measuring egocentric motion and body motion, proprioceptive sensors provide data when GPS, LiDAR, or external landmarks are unavailable. Among proprioceptive sensors, IMUs are common hardware that measures linear acceleration and angular velocity to estimate body orientation and motion. However, body-mounted IMU data alone is not sufficient for accurate position estimation (Frosi et al., 2023). In all dead reckoning methods, errors grow over time as small drift in each incremental motion estimate propagates to subsequent updates (Stuntz et al., 2016). For IMU-based localization, drift accumulation is amplified by sensor noise, impacts, and platform vibrations. As a result, directly integrating low-cost IMU data provides less accurate displacement estimates than estimates based on commanded displacements.

Since IMU-only dead reckoning is prone to drift, many legged robot localization methods combine IMU measurements with additional sensing methods, such as leg odometry or ground contact (see Table 1). For example, quadrupeds use joint encoders to determine leg positions for forward kinematic odometry (Reinstein and Hoffmann, 2013), while foot-mounted IMUs determine stance foot contacts and slips (Yang et al., 2023). In hexapod robots, joint force and torque sensors have been used for 6-DoF leg odometry, including roll and pitch stabilization (Görner and Stelzer, 2013), and fusion with leg kinematics and IMU measurements in an extended Kalman filter framework (Lubbe et al., 2015). These approaches show that leg sensing improves dead reckoning accuracy, but also requires additional hardware on multiple legs. For small, low-cost multi-legged robots, such limitation motivates dead reckoning methods that only use compact, body-mounted sensors.

**TABLE 1:** Survey of localization methods across platforms and terrains.

Ref.	Robot type	Joint encoders?	Other sensors	ML method	Worst-case terrain
Bloesch et al. (2013)	Quadruped	Yes	IMU	—	Indoor (flat)
Yang et al. (2023)	Quadruped	Yes	IMU (body + foot)	—	Outdoor (varying)
Nisticò et al. (2025)	Quadruped	Yes	IMU, LiDAR, GPS	—	Indoor (slippery)
Reinstein and Hoffmann (2013)	Quadruped	Yes	IMU, foot pressure	Linear regression	Indoor (foam)
Buchanan et al. (2021)	Quadruped	Yes	IMU, torque sensors	CNN, 1D-ResNet18	Indoor (slippery)
Youm et al. (2025)	Quadruped	Yes	IMU	Neural measurement network	Indoor (slippery)
Gu et al. (2024)	Quadruped	Yes	IMU, force sensors	Recurrent neural network	Simulation (solid, uneven)
Lubbe et al. (2015)	Hexapod	Yes	IMU, force sensors	—	Indoor (flat)
Görner and Stelzer (2013)	Hexapod	Yes	Torque sensors	—	Indoor (gravel)
Yang et al. (2022)	Rat	Yes	IMU	—	Indoor (flat)
Ojeda and Borenstein (2007)	Human	No	IMU	—	Indoor (3D)
Shi et al. (2019)	Human	No	IMU	—	Outdoor (solid, flat)
Liu et al. (2020)	Human	No	IMU	CNN, 1D-ResNet18	Indoor (flat)
This work	Hexapod	No	IMU	CNN	Outdoor (in sand)

IMU-only localization has been successful in tracking pedestrians (Hou and Bergmann, 2021; Bai et al., 2024; Ojeda and Borenstein, 2007; Shi et al., 2019) and small animals (Yang et al., 2022). In these studies, direct joint sensing may be uncomfortable or distracting for the users who are wearing the sensors, and thus unavailable. As a result, inertial dead reckoning is improved using step detection or zero-velocity updates during stance phases (Table 1). However, these studies have been primarily conducted on flat, rigid or indoor surfaces. On deformable terrain, stance and swing transitions are less abrupt, making step detection and zero velocity updates less reliable. Also, legged robots provide information about the commanded motion that are not available on pedestrian and animal tracking, giving the opportunity to estimate the difference between the commanded and actual displacement.

Thus, existing methods are most limiting in environments where legged robots are most useful. In remote environments, exteroceptive sensing such as GPS, markers, or visual features (Edwards et al., 2023; Colomer et al., 2022) may be degraded or unavailable. In wet, dirty, or hazardous environments, additional sensors on each leg increase hardware cost and ingress protection difficulty. In soft granular media (e.g., desert sand, snow) or dense vegetation, foot of the platform experiences sinkage, slip, and penetration, making conventional assumptions such as zero velocity updates or command kinematics methods unreliable.

Crab-like robots designed for benthic search (Graf et al., 2021; Gong et al., 2023; 2024) face similar terrain and sensing challenges that make low-complexity localization more important. Additional legs improve stability during water-land transitions and locomotion on soft substrates, but adding leg-mounted sensors increases complexity, cost, and waterproof requirements. Underwater operations further limit localization options: GPS is unavailable due to signal attenuation (Taraldsen et al., 2011), while vision is degraded due to turbidity and occlusion (Yuan et al., 2022; Cho and Kim, 2017). Doppler velocity logs or acoustic positioning systems may be too costly or bulky for small low-cost platforms. However, these crab-like robots do not require long-range navigation. Instead, they need to efficiently execute local search patterns around expected target locations, such as locations identified by surface scans (Billings et al., 2002). As a result, dead reckoning accuracy determines spacing between search passes, and larger localization errors require denser coverage, longer search time, and higher energy use. This motivates body-IMU based localization methods that reduce drift without adding additional sensors to each leg.

Granular and deformable substrates introduce localization errors since locomotion is strongly affected by sinkage, slip, and substrate-dependent drag. For the hexapod robot used in this study, foot sinkage is part of the locomotion strategy to improve stability on soft substrates (Graf et al., 2021). Therefore, the effect of sinkage should not be treated as negligible disturbance, and the resulting locomotion error should be accounted for in the dead reckoning algorithm. One approach is to model the foot-terrain interaction directly, but such models are computationally expensive and difficult to calibrate, especially for remote locations (Nishishita and Ishigami, 2025). These conditions also limit the capability of simulation-trained models (Youm et al., 2025), which require accurate modeling of substrate properties and foot-terrain interaction. Learned models reduce some of the modeling requirements associated with explicit terrain interaction models, but still assume sensing and state information that may be unavailable on small, encoder-free robots. Prior work had used learned models for robot control, including a model predictive control framework to reduce path tracking errors (Ostafew et al., 2014) and learning-based estimation algorithms that predict motion from IMU data (Buchanan et al., 2021; Liu et al., 2020). However, these approaches assume rich state information and detailed models that may not be available for minimally instrumented legged robot on deformable terrain. Another study used recurrent neural networks to learn per-leg contact location bias and fused it with a Kalman filter framework to reduce leg odometry translation error (Gu et al., 2024), but still requires leg-embedded sensing.

The above studies demonstrate the value of learned inertial proprioceptive information, but focus on direct motion estimation and sensor-rich leg odometry rather than command-execution error under encoder-free sensing constraints. Motivated by these limitations, we investigate whether measurements from body-mounted IMUs and commanded motion provide displacement-bias correction for encoder-free legged dead reckoning. Our approach is based on the hypothesis that, despite sensor noise, body-frame acceleration profiles contain motion signatures, defined here as repeatable acceleration patterns, that are associated with gait execution and substrate interaction. These signatures correspond to command-to-displacement bias when the realized body displacement differs from the commanded displacement. Instead of using learned inertial-odometry methods that estimate motion directly from sensor measurements, the proposed method retains commanded body displacement as a nominal estimate and predicts the residual command-to-displacement bias for each two-step gait cycle. We train a single CNN bias estimator on a combined indoor foam and sand dataset. The estimator predicts platform-specific per-cycle displacement bias using commanded motion and IMU acceleration profiles without requiring joint encoders, contact sensing, explicit terrain labels, classification, or model switching. The contribution of this approach is therefore a sensor-minimal, command-to-displacement bias correction framework for an encoder-free hexapod operating on deformable terrain, instead of a general learned inertial-odometry method.

We evaluate this approach on an encoder-free 18-DoF hexapod walking on soft and granular substrates. The robot carries a rigidly mounted four-IMU array - the maximum number accommodated by the available mounting space, where the filtered outputs are averaged for heading correction and CNN-based displacement bias correction. Repeated outdoor field tests compare naive, heading corrected, and heading + neural network corrected dead reckoning. The results show that the proposed correction method reduces mean dead reckoning drift, root mean square error (RMSE), and total drift relative to the idealized kinematic dead reckoning baseline. The improvement is especially noticeable on sand, where terrain dependent sinkage introduces systematic displacement undershoot during encoder-free locomotion.

This approach is not intended to replace encoder-based odometry or extended Kalman filter frameworks on sensor-rich platforms. Instead, it provides a compact dead reckoning drift reduction method for minimally instrumented legged platforms performing local searches on deformable, irregular terrain. By correcting for systematic displacement errors from body-mounted IMU signatures, drift is compensated from a desired search path without additional sensors on each leg.

## 2 Methods

### 2.1 Encoder-free hexapod platform & sensing constraints

**FIGURE 1:**
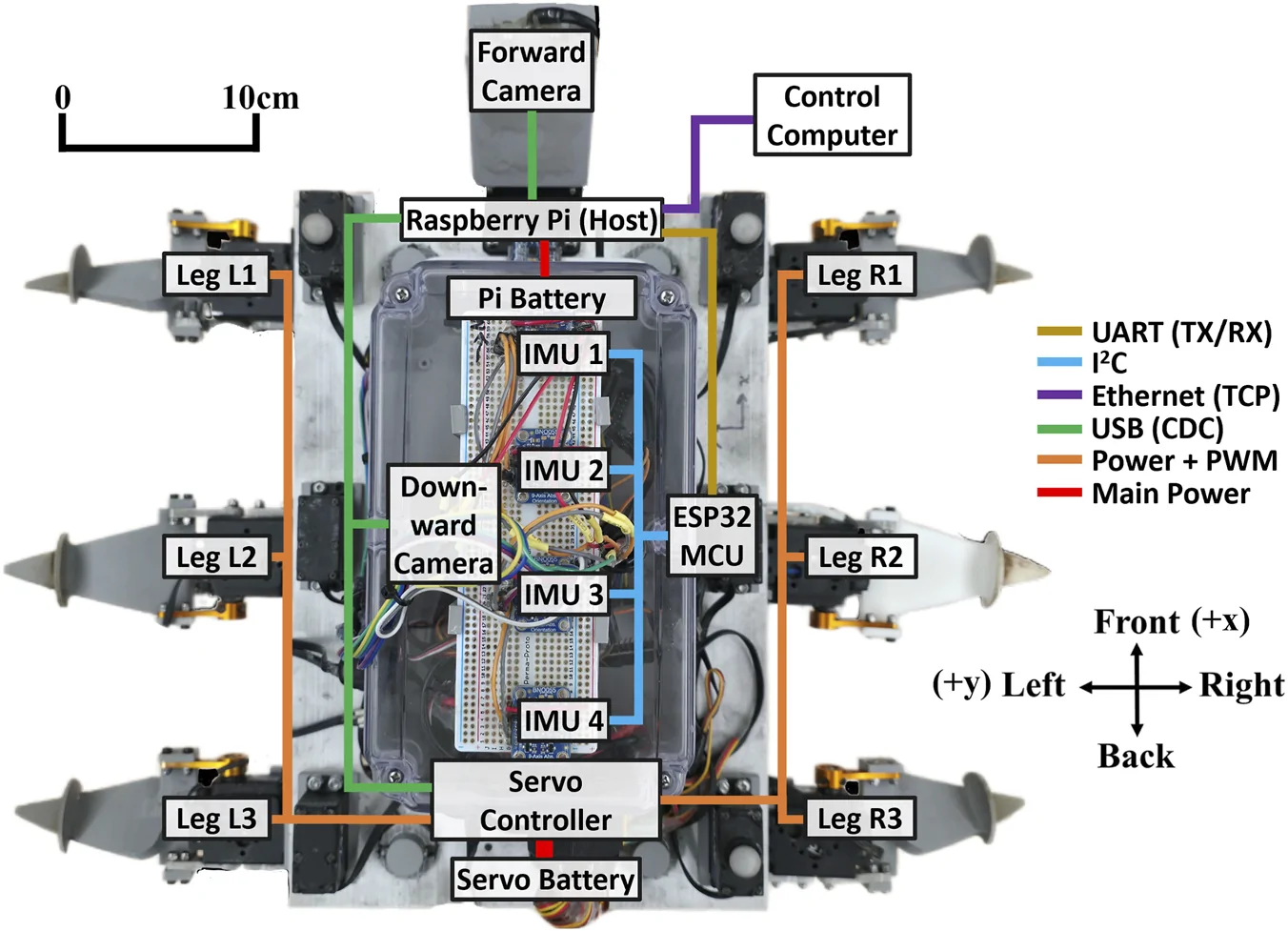
Hexapod hardware layout and multi-IMU array. The top-down schematic shows four added IMUs, onboard processing modules, and communication links.

An encoder-free 18-DoF hexapod robot (Figure 1) is used as the experimental platform, with three-DoF per leg actuated by Savox SW2210SG servos. The servos are powered by a 7.4 V Li-Po battery and controlled by a Pololu Maestro 18-Channel USB controller. The platform does not include any joint encoders or contact sensors, which reflects the original design constraints based on size, cost, and robustness of operation on granular media in the previous study (Gong et al., 2024). The proposed dead reckoning approach is therefore designed for this minimally instrumented hexapod under terrain conditions where conventional encoder-based odometry assumptions are hard to maintain. An additional $150 of sensing upgrades is added, increasing the total cost of the robot to approximately $2,450.

To enable real-time control, responsive teleoperation, and synchronized data logging during field experiments, the robot uses an onboard-offboard communication architecture. A Raspberry Pi 5B functions as a real-time I/O and networking hub, hosting a Transmission Control Protocol (TCP) server, executing servo locomotion commands, and streaming parsed IMU data from an ESP32 microcontroller and compressed video streams from forward- and downward-facing cameras. The Pi connects to a control computer via Ethernet, where a multithreaded GUI client establishes the TCP link, generates and transmits search patterns and locomotion commands, and logs and visualizes robot state data. The teleoperation pipeline achieves stable performance, with 50–60 m round-trip latency, downstream throughput of 12–13 Mb/s for dual video and IMU data streams, and upstream command packet transmission of at least 48 bytes.

### 2.2 Multi-IMU hardware & sensing

A rigidly mounted IMU array consisting of four BNO055 9-DoF absolute orientation sensors is mounted 2.8 cm above the enclosure to measure accelerometer, gyroscope, and magnetometer data. Prior study on multi-IMU experiments had shown that navigation RMS errors decrease as the number of IMUs increases, with diminishing improvements and increasing power consumption (Larey et al., 2020). Using the empirical expressions fitted by (Larey et al., 2020) to their stationary data, increasing the number of IMUs from one to four theoretically reduces position and attitude RMSE by approximately 49.4% and 40.2% respectively. The available mounting area of 19.3 cm by 11.7 cm accommodates a maximum of four IMUs without increasing platform dimensions. The array aims to reduce uncorrelated sensor noise and sensor-specific bias in measurements supplied to the roll, pitch, and yaw estimation algorithm as well as the CNN bias estimator. Therefore, the IMU array configuration in Figure 1 is chosen to maximize separation between sensors within the available mounting area. An ESP32 operates as a dual-I2C host that enables synchronous data collection from sensors with identical addresses.

Before each experiment, each IMU is required to reach full internal calibration status using the IMU’s onboard calibration routine. The robot is then placed on a flat stationary surface, and a stationary bias estimate is calculated independently for each IMU. The gyroscope, accelerometer and linear acceleration biases are estimated by averaging samples collected during the stationary interval. The calculated bias terms are subtracted from subsequent measurements from the corresponding sensor. After bias compensation, each measurement channel from each IMU was first passed through a first-order low-pass filter (α=0.5) to filter high-frequency noise. Each channel is then processed using an independent scalar Kalman filter to further reduce measurement noise. Filtered measurements from the four IMUs are then fused using an equal-weight arithmetic mean. Under the assumption of equal-variance sensor noise, the standard deviation of the measurement is $\sigma_{N}=\sigma_{1}/\sqrt{N}$ (Larey et al., 2020), where N is the number of sensors. Averaging four sensors theoretically reduces random noise standard deviation by 50% relative to a single sensor. The averaged gyroscope, accelerometer, and magnetometer measurements are used to estimate roll, pitch, and yaw. Roll and pitch are calculated using a complementary filter that combines gyroscope integration and accelerometer inclination estimates. The yaw angle (heading) is calculated from magnetometer measurements after compensating for body roll and pitch. The ESP32 implements a multi-IMU fusion pipeline and streams roll, pitch, yaw, accelerometer data, and gyroscope data to the Pi at 60–65 Hz.

### 2.3 CNN per-cycle displacement bias correction

The CNN bias estimator is constructed based on the hypothesis that body-mounted IMU acceleration profiles contain motion signatures associated with platform-substrate interaction. During each two-step gait cycle, measured accelerations are influenced by factors such as stance loading, foot sinkage, touchdown impact, and body oscillations. Although these measurements include sensor noise and gait-induced disturbances, they may capture changes when the realized displacement differs from the commanded displacement on deformable terrain.

The CNN bias estimator predicts planar displacement bias for each two-step gait cycle, where the displacement bias is defined as the difference between the commanded body displacement and true body displacement obtained from the motion captured ground truth for training. As a result, the model is trained as a platform- and gait-specific command-to-displacement bias estimator.

As the robot walks, body-frame linear acceleration $a_{x}\left(t\right),a_{y}\left(t\right)$ are recorded from the IMU array. The acceleration component in the direction of the commanded displacement, $a_{t}$ , is extracted:

$$a_{t}\left(t\right)=\frac{a_{x}\left(t\right)\cdot \Delta x_{i}+a_{y}\left(t\right)\cdot \Delta y_{i}}{\sqrt{\Delta x_{i}^{2}+\Delta y_{i}^{2}}}$$ (1)

The acceleration component along the commanded direction is used, since the observed dominant displacement error is consistent displacement undershoot along the commanded direction, and the robot has negligible heading change over the two-step gait cycle. The lateral and vertical acceleration components are not used in the bias estimator, which keeps it compact and reduces its sensitivity to gait-induced oscillations that are less directly associated with command displacement undershoot. Equation 1 is evaluated for two-step gait cycles with nonzero translational commands. If the waypoint distance is below the 2.5 cm stopping threshold, no translation command is issued. Pure rotations are excluded as the platform turns in place about its body center, and heading is updated separately using IMU-derived yaw. The retained time series data are rescaled across 250 samples using linear interpolation.

The processed acceleration data are passed through a one-dimensional convolutional layer with a kernel size of 40 and five output channels to extract features. The features are then concatenated with the commanded body displacement $\left(\Delta x_{i},\Delta y_{i}\right)$ and processed through three fully connected layers with output size 250. A final fully connected layer outputs a 2 × 1 vector $\left({\hat{\varepsilon }}_{xi},{\hat{\varepsilon }}_{yi}\right)$ that predicts the estimated command-to-displacement bias (realized displacement minus commanded displacement). The CNN is selected based on the fixed-window, per-cycle nature of the input data. Each bias estimate is generated using acceleration profiles recorded during a fixed-duration, two-step gait cycle to capture patterns associated with gait-substrate interactions. Since each cycle is processed independently, longer-term dependencies handled by LSTM is not modeled. Compared with a shallow CNN used here, the 1D-ResNet18 architecture is substantially deeper with greater capacity. The CNN architecture is therefore chosen for its compactness and compatibility with per-cycle estimation.

The CNN bias estimator is constructed using the PyTorch library (Ansel et al., 2024) and trained using a mean-squared-error (MSE) loss function between the predicted and true displacement error, using the Adam optimization algorithm (Kingma and Ba, 2015), with a learning rate of $5\times 10^{-5}$ and a batch size of 64 for 200 epochs. 20 estimators are trained using these parameters. The best-performing estimator is selected by comparing dead reckoning accuracy on a separate model selection trial set, consisting of ten path-following trials on grass and sand (totaling approximately 600 two-step gait cycles). The model selection trial set is not included in outdoor evaluation described in Section 3. After the best estimator is identified, its parameters are fixed and integrated into the robot’s dead reckoning algorithm without terrain classification or model switching. This design examines whether an estimator trained across multiple indoor flat terrain conditions transfers to an outdoor substrate that is not represented in the training dataset.

**FIGURE 2:**
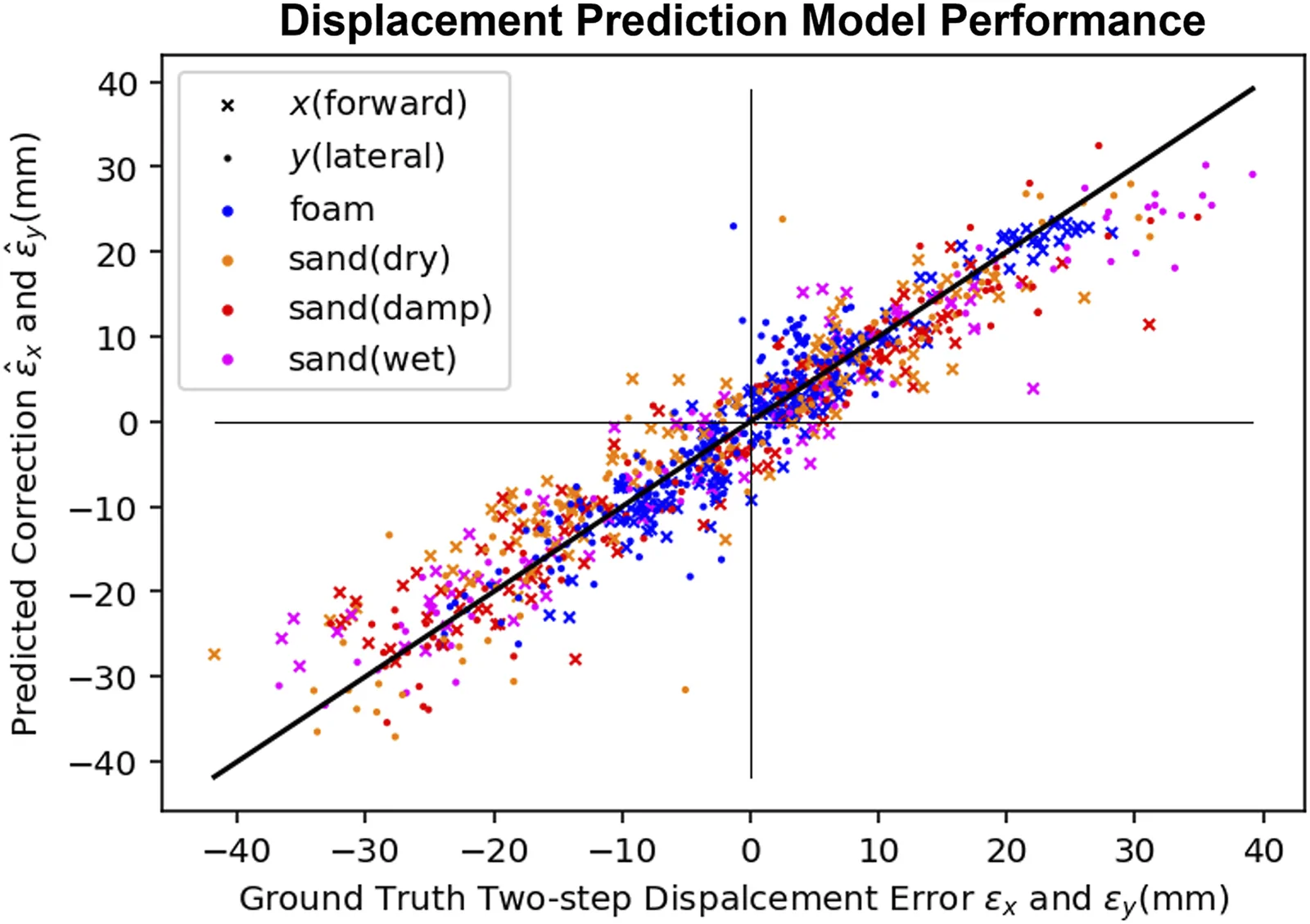
Accuracy of the per-cycle displacement prediction model on the validation set. Each point reflects the forward (x) or lateral (y) component of motion of a two-step gait cycle. The true difference between the robot’s motion and the commanded displacement is plotted on the horizontal axis and the model’s prediction on the vertical.

To determine whether acceleration profiles provide additional information compared to a fixed command displacement correction, the CNN is compared with the uncorrected commanded displacement and a least-squares linear regression model, mapping from commanded to actual displacements. The estimated command-to-displacement biases for the validation dataset are plotted against the true values in Figure 2. The RMS error of the CNN bias estimator on the validation set is 6.70 mm, while the RMS error between the commanded and true displacements is 20.9 mm. The coefficients of determination of x and y components are $R^{2}=0.89\text{ and }0.90$ , respectively. A linear function of the form

$$A\left[\begin{array}{c} \Delta x_{i}\\ \Delta y_{i} \end{array}\right]=\left[\begin{array}{c} \Delta x_{i}+\varepsilon_{xi}\\ \Delta y_{i}+\varepsilon_{yi} \end{array}\right]$$

is fit to the recorded data, which compensates for systematic errors. A least-squares solution calculated for the training dataset results in an RMS error of 10.6 mm when applied to the validation set. If the training data is segmented by terrain type to account for terrain-specific bias, individual linear models produce RMS errors of 7.73 mm for foam and 8.53 mm for sand. As a result, the CNN reduced validation RMS error by approximately 67.9% compared to commanded displacement alone, and 36.8% compared to the linear regression baseline. The CNN also outperformed terrain-specific linear regression models while being one combined model without terrain labels.

### 2.4 Encoder-free planar dead reckoning

The robot performs one planar dead reckoning update after each two-step gait cycle. After each dead reckoning update, the body-frame displacement is transformed to the world frame using the robot’s heading (yaw angle) about the vertical axis. Roll and pitch are estimated by the multi-IMU fusion pipeline for tilt-compensated heading estimation, but are not used to project a full three-dimensional displacement vector in the world frame. This planar projection is consistent with the scope of the experiments, where supervised training data are collected on relatively flat indoor foam and sand surfaces, and outdoor validation trials are conducted on relatively flat grass and sand terrain. Therefore, under the tested terrain conditions, roll and pitch induced projection errors are secondary relative to heading drift and command-to-displacement bias.

In this work, three dead reckoning algorithms are tested with increasing utilization of sensor data. The first algorithm, naive dead reckoning, does not utilize onboard IMU measurements and relies only on the commanded body displacement generated by the gait controller. The dead reckoning position estimate $\left(\hat{X},\hat{Y}\right)$ and orientation $\hat{\theta }$ after N completed two-step gait cycles are:

$$\left[\begin{array}{c} {\hat{X}}_{N}\\ {\hat{Y}}_{N} \end{array}\right]=\left[\begin{array}{c} X_{0}\\ Y_{0} \end{array}\right]+\overset{N}{\underset{i=1}{\sum }}\left(R_{i}\left[\begin{array}{c} \Delta x_{i}\\ \Delta y_{i} \end{array}\right]\right)$$ (2)

$$R_{i}=\left[\begin{array}{cc} \cos{\hat{\theta }}_{i} & -\sin{\hat{\theta }}_{i}\\ \sin{\hat{\theta }}_{i} & \cos{\hat{\theta }}_{i} \end{array}\right]$$ (3)

$${\hat{\theta }}_{N}=\theta_{0}+\overset{N}{\underset{i=1}{\sum }}\Delta \theta_{i}$$ (4)

where $\left(X_{0},Y_{0}\right)$ and $\theta_{0}$ are the robot’s initial planar position and orientation. $\left(\Delta x_{i},\Delta y_{i}\right)$ and $\Delta \theta_{i}$ are the commanded displacement and rotation of the i th two-step gait cycle. Since this approach lacks feedback, it represents the idealized motion of the platform that would occur if each command is executed exactly. Therefore, deviations caused by servo backlash and dactyl sinkage are not accounted for, causing dead reckoning error to accumulate rapidly over time, a phenomenon known as drift. Drift in heading is particularly detrimental because a discrepancy between θ and $\hat{\theta }$ induces additional error in $\hat{X}$ and $\hat{Y}$ with each two-step gait cycle.

To reduce heading drift, the second algorithm, termed heading correction (HC), uses IMU-derived yaw estimate directly in each dead reckoning update. Dead reckoning is performed as above, except ${\hat{\theta }}_{i}$ is measured directly and used to update Equation 4. At the beginning of each cycle, a backward moving average filter with a width of 30 samples (approximately 0.5 s) is applied to heading measurements to smooth out oscillations that occur within a two-step gait cycle and provide a more stable heading estimate. This approach takes advantage of the Earth’s magnetic field as an absolute orientation reference and is resistant to heading drift. However, HC does not correct for translation errors that occur when realized displacement differs from the commanded displacement, and dead reckoning drift still accumulates due to terrain-induced displacement undershoot.

The third algorithm, heading correction with neural network (HC + NN), improves the accuracy of HC by implementing a CNN per-cycle displacement bias correction. The bias estimator described in Section 2.3 processes body-frame linear acceleration measurements from each two-step gait cycle. Estimated command-to-displacement bias outputs $\left({\hat{\varepsilon }}_{xi},{\hat{\varepsilon }}_{yi}\right)$ are used to modify Equation 2. Equation 5 gives a corrected dead reckoning position estimate using the same rotation matrix of Equation 3:

$$\left[\begin{array}{c} {\hat{X}}_{N}\\ {\hat{Y}}_{N} \end{array}\right]=\left[\begin{array}{c} X_{0}\\ Y_{0} \end{array}\right]+\overset{N}{\underset{i=1}{\sum }}\left(R_{i}\left[\begin{array}{c} \Delta x_{i}+{\hat{\varepsilon }}_{xi}\\ \Delta y_{i}+{\hat{\varepsilon }}_{yi} \end{array}\right]\right)$$ (5)

### 2.5 Path-following

To enable systematic area search, the robot follows a pre-generated search pattern. The pattern is generated by defining a sequential set of waypoints $\left(X_{j}^{*},Y_{j}^{*}\right)$ that represent the desired robot path in a fixed reference frame and a desired heading $\theta^{*}$ to maintain. The GUI transmits the path to the robot’s onboard computer through a TCP packet.

**FIGURE 3:**
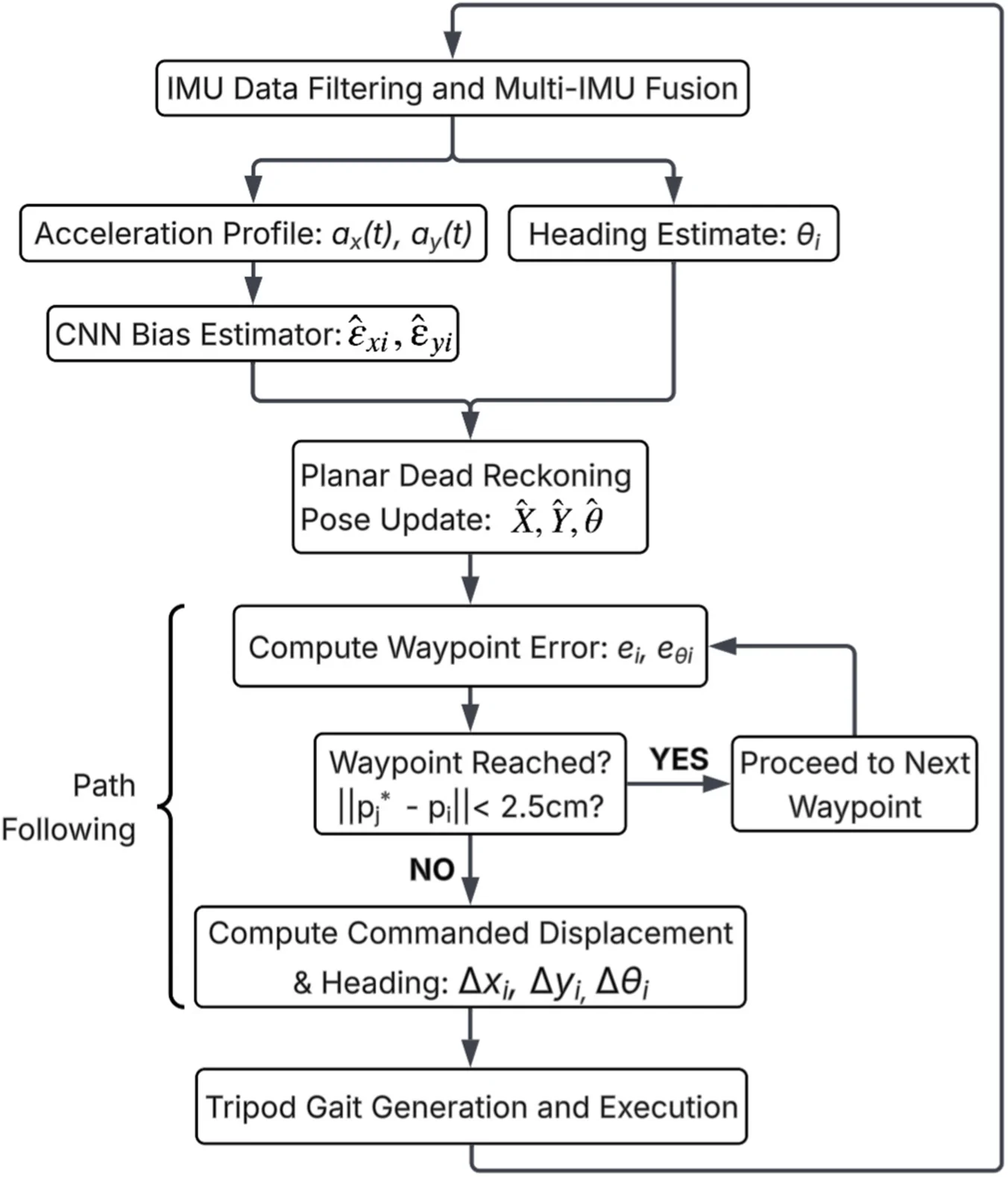
Multi-IMU aided planar dead reckoning pipeline. IMU derived heading and CNN command-to-displacement bias correction are combined in the planar pose update. The update is then used for waypoint-based path-following and tripod gait execution.

The robot traverses the path by traveling to consecutive waypoints, starting with j=1 , in a series of discrete two-step gait cycles. The path-following algorithm is visualized in Figure 3. Before each cycle, the robot updates its dead reckoning position estimate $\left(\hat{X},\hat{Y},\hat{\theta }\right)$ (Section 2.4). Equation 6 then calculates the error between the waypoint and its current position estimate, expressed in the robot’s local reference frame:

$$\begin{array}{ccc} & e=\left[\begin{array}{cc} \cos{\hat{\theta }}_{i} & \sin{\hat{\theta }}_{i}\\ -\sin{\hat{\theta }}_{i} & \cos{\hat{\theta }}_{i} \end{array}\right]\left[\begin{array}{c} X_{j}^{*}-{\hat{X}}_{i}\\ Y_{j}^{*}-{\hat{Y}}_{i} \end{array}\right], & e_{\theta }=\theta^{*}-{\hat{\theta }}_{i} \end{array}$$ (6)

If the magnitude of the error ‖e‖<10 cm (our pre-defined maximum commanded displacement), the commanded displacement for the next two-step gait cycle is $\left(\Delta x_{i},\Delta y_{i}\right)=e$ , otherwise $10\text{ cm}\frac{e}{\left\|e\right\|}$ . Similarly, $\Delta \theta_{i}=e_{\theta }$ if $|e_{\theta }|\leq 30{}^{\circ}$ . The robot executes one two-step gait cycle using these commanded displacement parameters, then repeats the process until the estimated position error is below a threshold of 2.5 cm, at which point it proceeds to the next waypoint (incrementing j ).

### 2.6 Tripod gait generation and execution

**FIGURE 4:**
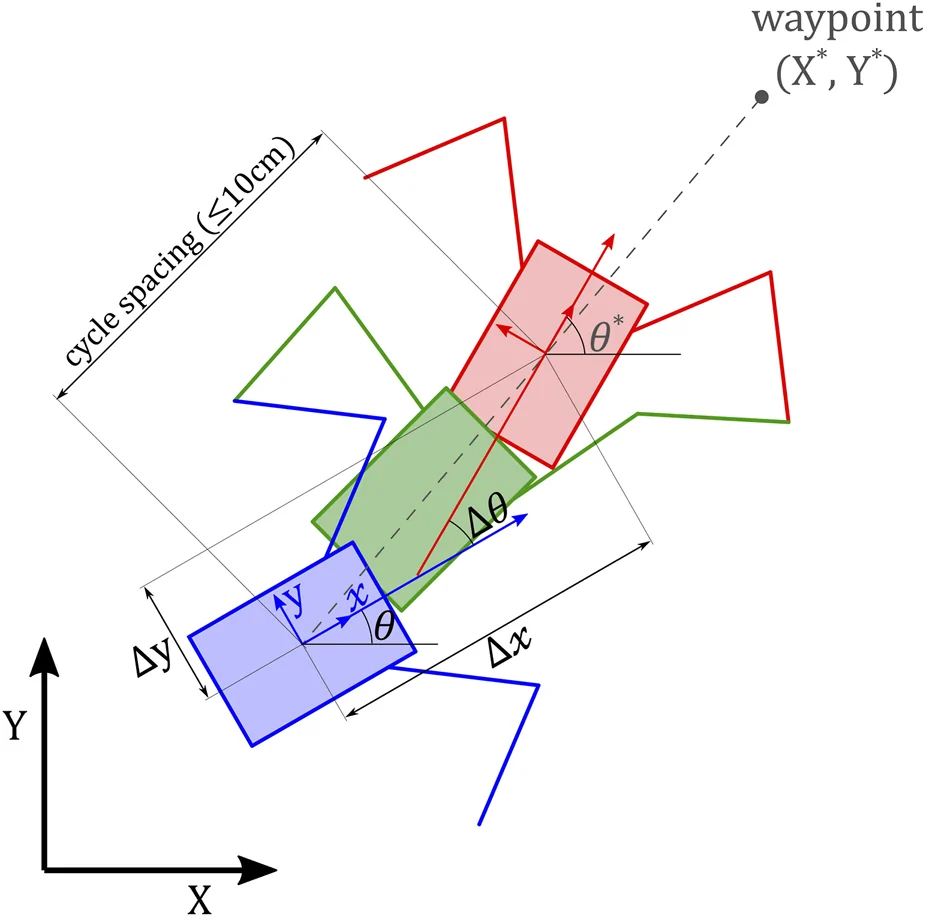
A two-step gait cycle showing the motion of two legs. The robot’s initial state is shown in blue, the intermediary state after one step in green, and its final state after two steps in red. The other four legs, not shown, move similarly.

The robot uses an alternating tripod gait for all experiments, as shown in Figure 4. The gait is selected as it supports rapid traversal while maintaining a triangular support polygon, making it appropriate for waypoint-based local search tasks considered in this study. The end-effectors (tips of the dactyls) follow a trajectory that consists of four phases: stance, lift, swing, and descent. During the first half of each cycle, tripod 1 (legs L1, R2 and L3) stance while tripod 2 (legs R1, L2 and R3) swing forward. In the second half, the tripod roles are reversed. For a commanded body displacement $\left(\Delta x_{i},\Delta y_{i}\right)$ and rotation $\Delta \theta_{i}$ , each half-cycle advances and rotates the body by $\left(\Delta x_{i}/2,\Delta y_{i}/2\right)$ and $\Delta \theta_{i}/2$ at constant speed.

The end-effector trajectories are calculated to produce the desired body displacement assuming no slipping of the dactyl tip. We consider a stationary global reference frame, W , and a fixed robot body-frame, B , coincident at time t=0 . The position of an end-effector in each frame at time t is denoted by pw(t) and pB(t) , respectively. The desired pose of the body relative to the global frame is expressed as a transformation matrix $g_{BW}\left(t\right)$ . During the stance phase, each end-effector is assumed to be stationary in W while the body follows the desired trajectory. Under this condition, the trajectory of each end-effector with respect to the robot body during the stance phase is determined by its initial position:

pB(t)=gBWt⋅pw0 (7)

We impose a continuity constraint on the trajectory by setting _*W*_ p(0) in Equation 7 to equal to the end-effector position at the end of the previous gait cycle. During the swing phase, the dactyl lifts 45 mm vertically to clear sand or small obstacles, swings horizontally, then plants straight down. The touch down position is controlled by the horizontal displacement in the swing phase.

The resulting trajectories are converted into joint angles using inverse kinematics described in section II.C of (Gong et al., 2024). If a joint angle exceeds its defined mechanical range limit (which tends to occur when the robot changes direction while taking large displacement commands), the commanded displacements $\Delta x_{i}$ , $\Delta y_{i}$ , and $\Delta \theta_{i}$ are reduced by 25% and the gait trajectory is recalculated.

### 2.7 Training data for walking error characterization

Training data for the CNN bias estimator are collected indoors by tracking the robot walking on foam tiles and on sand with three moisture content, where higher moisture levels reduce dactyl sinkage but increase drag during dactyl extraction. Ground truth data are recorded (Section 2.8) and synchronized with onboard accelerometer data and gait cycle displacement commands. The commanded body displacement, true displacement, and measured acceleration profile associated with each two-step gait cycle are extracted. Outliers that exceed 50 mm of measured displacement error (possibly caused by errors in the tracking system) are discarded from training.

On foam tiles, the robot executes 30 full-size (10 cm) two-step gait cycles and 30 half-size (5 cm) cycles in each of four directions (left, right, forward, and backward), followed by three spiral pattern walks, adding an additional 297 cycles in various directions. The 10 cm and 5 cm displacement commands are executed using the same gait cycle of 1.8 s, representing two nominal speeds. The two displacements provide training data for full length commands used during the majority of waypoint traversal and shorter commands during adjustments of the final waypoint.

In sand, limited space required a different approach: for each of three moisture content levels, the robot takes nine 10 cm two-step gait cycles forward, followed by nine in the opposite direction. This is repeated, varying the direction of travel with respect to the robot body in 15° increments from 0° to 345° . The same process is used for 5 cm displacements with 30° increments in sand with two moisture content levels, which improves generalization of the bias estimator on granular terrain. In total, 1,414 two-step gait cycles (approximately 42 min of walking) with varying step sizes and directions are recorded across different terrains and conditions, of which 8 are rejected as outliers. The retained cycles are randomly split at individual two-step gait cycle level, with 70% selected to form a training dataset and 30% used for validation. Explicit terrain labels are not provided to the model.

**FIGURE 5:**
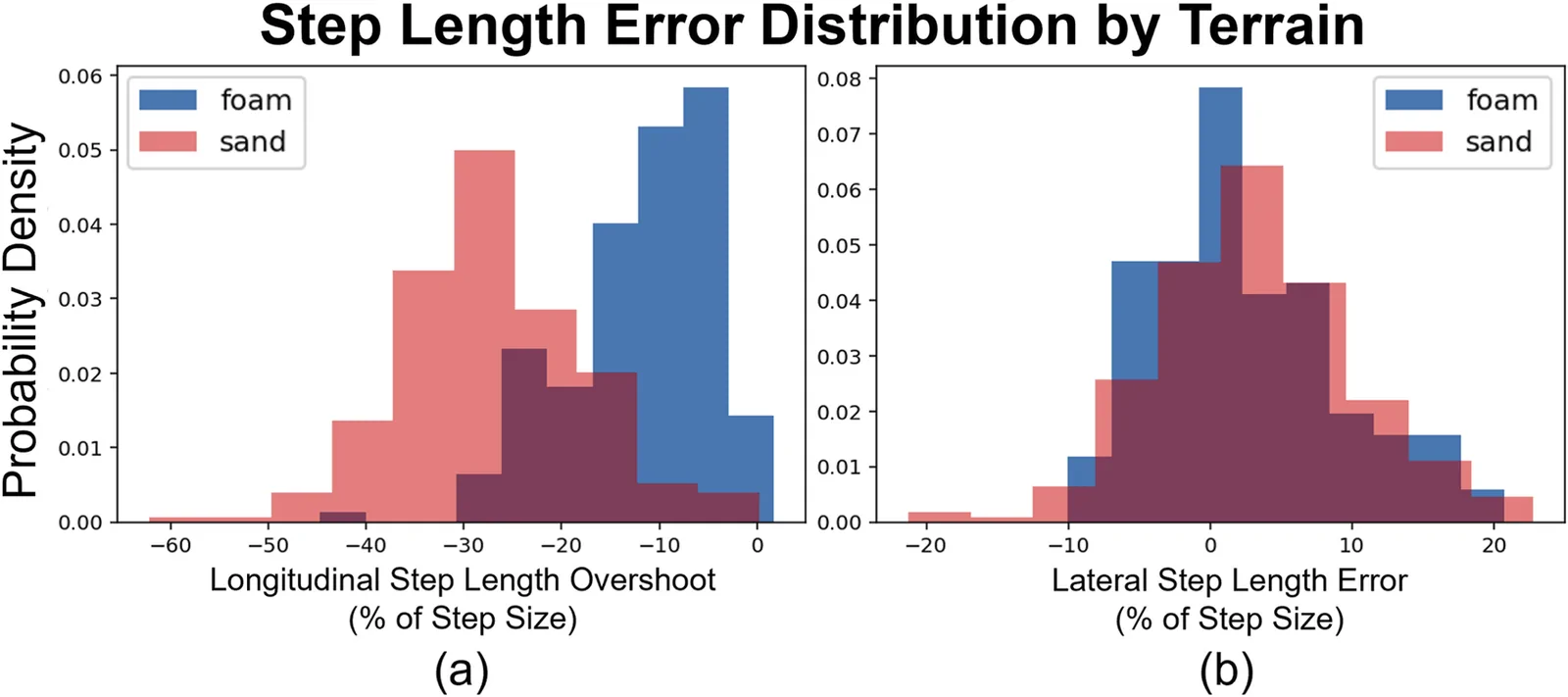
Step error distribution as a percentage of commanded step length. **(a)** Overshoot is the component parallel to the commanded displacement. **(b)** Lateral error is the orthogonal component.

Figure 5 shows the measured distribution of displacement errors. When walking on foam, the forward displacement undershoots the commanded length, generally between 5% and 15%. On sand, the undershoot increases to roughly 20%–35% due to dactyl sinkage. The estimator approximates this command-to-displacement bias without explicit knowledge of the terrain.

### 2.8 Ground truth position data collection

Two external tracking systems are used for different purposes of the study.

For supervised training of the CNN bias estimator, high accuracy training data is required for each two-step gait cycle. A Vicon Vero motion capture system is used in an indoor lab environment to track three reflective markers attached to the robot, giving three-dimensional body position and orientation. The Vicon system has a mean accuracy of at least 0.02 mm according to manufacturer specifications (Vicon Motion Systems Ltd, 2026).

For recording the platform’s true position during field tests, an overhead camera with fixed resolution is used. Tracker, a video analysis tool maintained by the Open Source Physics Project (Christian et al., 2026), is used to track the position of the center of the robot in two dimensions. In a lab validation test, the average discrepancy between the overhead camera and the Vicon motion capture system was found to be approximately 2.3 cm. The overhead camera’s accuracy is sufficient for evaluating dead reckoning drift during outdoor path-following trials, but is not sufficient for generating per-cycle supervised training data. Therefore, all training data for the bias estimator are collected indoors using Vicon on foam and sand, while the overhead camera is used only for outdoor trajectory evaluation.

## 3 Experiments and results

### 3.1 Experimental setup

**FIGURE 6:**
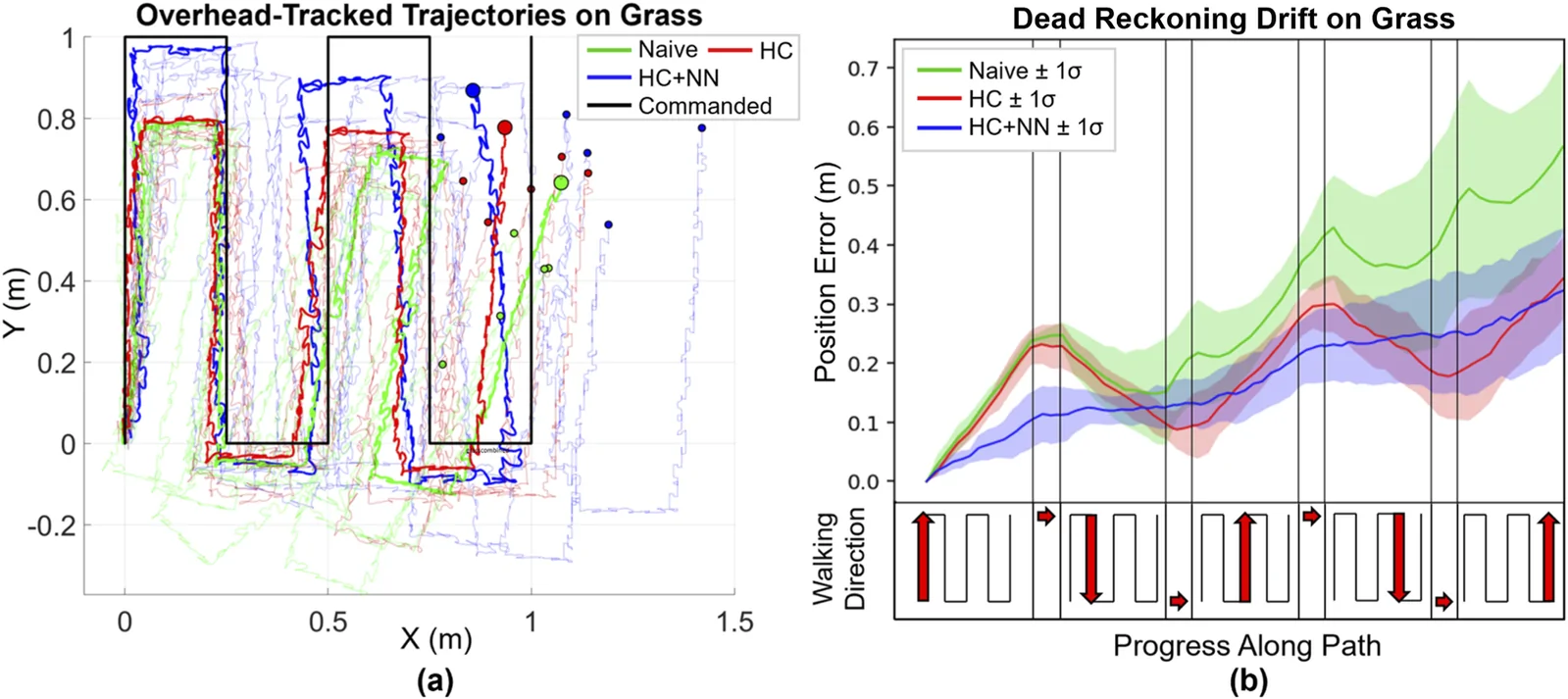
**(a)** Robot trajectories tracked using an overhead camera on grass. The commanded trajectory is shown in black, and the highlighted trajectories represent trials with the lowest mean drift from the commanded pattern. **(b)** Dead reckoning drift over time averaged across trials. The horizontal axis is number of two-step gait cycles, normalized by the total number of cycles in each trial.

**FIGURE 7:**
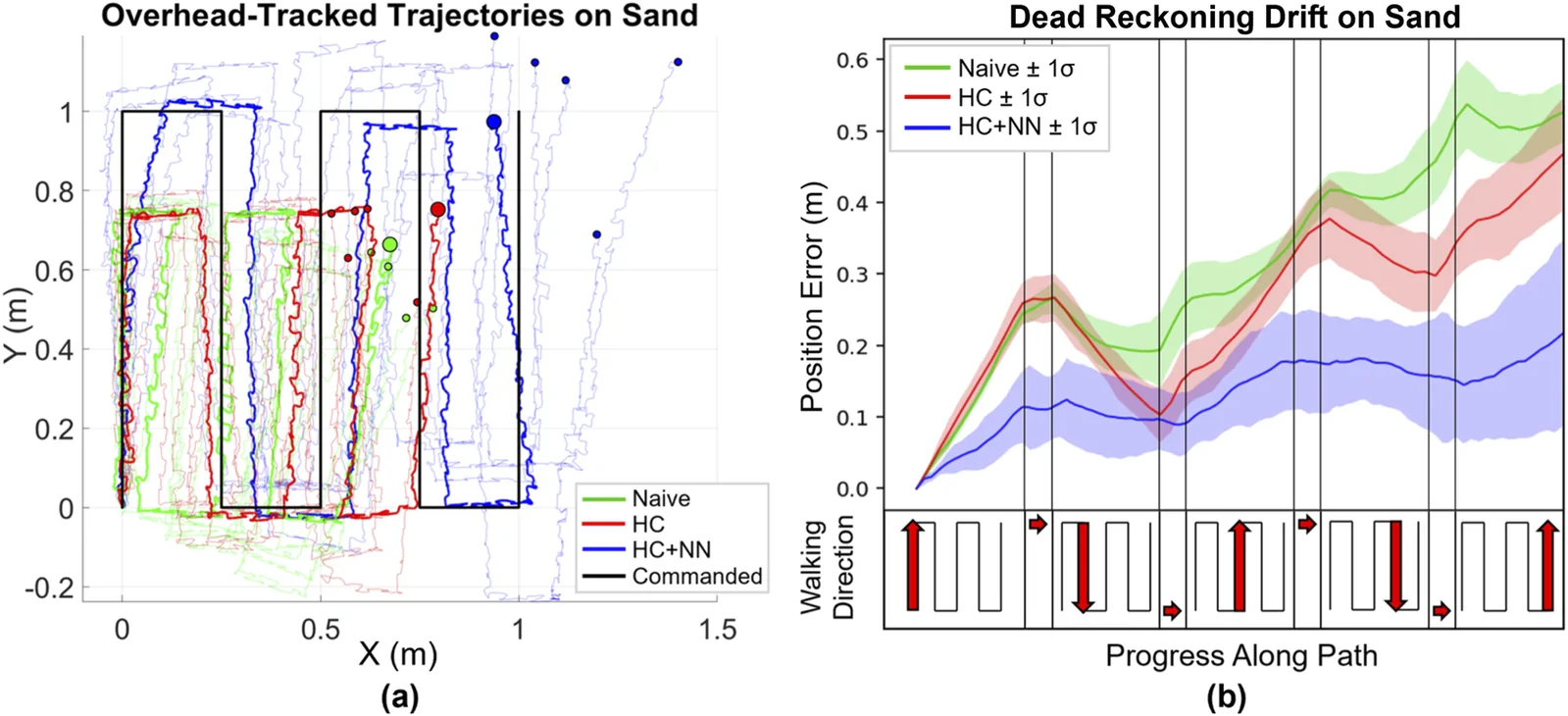
**(a)** Robot trajectories tracked using an overhead camera on sand. The commanded trajectory is shown in black, and the highlighted trajectories represent trials with the lowest mean drift from the commanded pattern. **(b)** Dead reckoning drift over time averaged across trials. The horizontal axis is number of two-step gait cycles, normalized by the total cycles in each trial.

To compare the three dead reckoning algorithms, six trials using each algorithm were conducted on a grassy field and an outdoor sand pit with 10 inches depth. A 1 m × 1 m ladder search pattern with 0.25 m step-over was used for all trials, as shown in black in Figures Figures 6a, 7a. This pattern provides systematic area coverage and is commonly used in search-and-rescue operations (Thomas, 2013). The starting position and orientation of the robot were changed between trials to minimize the effect of local differences in surface slope and density. The robot’s true position at each timestamp was obtained from the overhead camera tracking data described in Section 2.8.

### 3.2 Comparisons between naive dead reckoning, heading correction (HC), and HC + neural network (HC + NN)

The robot’s true position and dead reckoning drift over time are shown in Figure 6 for grass and Figure 7 for sand. Shaded regions represent the standard deviation between trials for each configuration.

**FIGURE 8:**
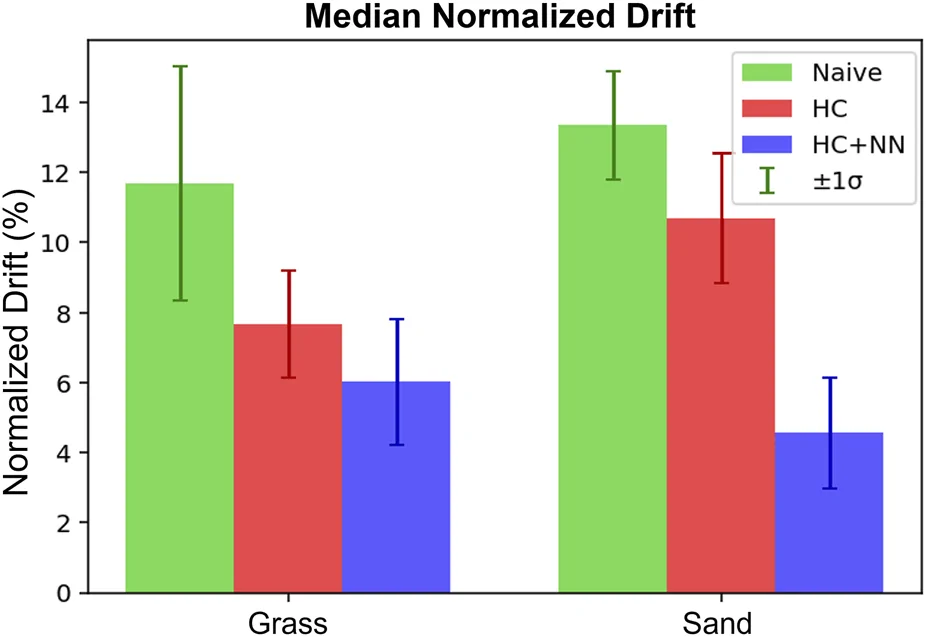
Localization algorithm drift comparison. Each bar shows the average error across six trials.

Instantaneous position error is defined as the Euclidean distance between the robot’s true position and its dead reckoning position estimate, recorded after each two-step gait cycle. For each trial, mean dead reckoning drift, RMSE (square root of mean squared dead reckoning drift), and total drift (distance between the final true position and the final dead reckoning estimate) are calculated. Additionally, normalized drift is defined as the ratio of instantaneous position error divided by the cumulative distance traveled. For each pairing of substrate and dead reckoning algorithm, per-trial metrics are averaged, with standard deviation calculated across trials (Table 2). Median normalized drift throughout a trial, shown in Figure 8, is taken as a measure of dead reckoning drift.

**TABLE 2:** Comparison of path tracking performance.

Absolute performance	Grass			Sand		
	Naive	HC	HC + NN	Naive	HC	HC + NN
Mean drift (cm)	29±7.8	19±4.1	17±5.2	32±2.8	26±3.7	13±5.1
RMSE (cm)	33±9.0	21±4.4	19±6.1	35±3.0	28±4.0	15±5.4
Total drift (cm)	59±15	36±7.1	33±11	53±4.1	49±8.3	22±13
Median normalized drift (%)	12±3.3	7.7±1.5	6.0±1.8	13±1.6	11±1.8	4.6±1.6

### 3.3 On outdoor grass

The HC configuration improves dead reckoning performance on uneven terrain compared to the naive baseline, where mean drift, RMSE, and total drift are reduced by 34%, 36% and 38%, respectively. The HC + NN configuration reduces mean drift, RMSE, and total drift by 11%, 10% and 8% compared to HC. HC reduces median normalized drift on grass from 12% to 7.7%, while the addition of CNN displacement bias correction further reduces it to 6.0% (Figure 8, Grass).

### 3.4 On outdoor sand

Compared to the naive baseline, HC reduces mean drift, RMSE, and total drift by 18%, 18% and 9%. The CNN displacement bias correction further improves dead reckoning performance compared to HC, with approximately 50% reduction in mean drift and RMSE, and 54% reduction in total drift. Median normalized drift also reduces from 13% (naive) to 11% (HC) to 4.6% (HC + NN) (Figure 8, Sand). The applied correction reduces dead reckoning drift overall, even though the corrected trajectories show greater trial-to-trial variability.

## 4 Discussion

The terrain-dependent improvements of HC and HC + NN highlight both the dominant source of dead reckoning drift and the importance of similarity between training and deployment conditions.

On grass, heading correction provides the largest improvement relative to the baseline, while CNN displacement bias correction provides a lower additional improvement. This indicates that heading error is a dominant source of dead reckoning drift on the tested grass surface. The non-uniform grass layer has variations in surface stiffness and local depressions, which may cause variable dactyl penetration depth, intermittent slip, and asymmetric contact forces. These effects may generate yaw drift rather than repeatable command displacement undershoot that is corrected by the CNN bias estimator. The limited additional improvement of HC + NN is also consistent with the construction of the training data, which does not include outdoor grass. Therefore, the bias estimator had to generalize to acceleration signatures that are not fully represented in the training dataset.

On sand, the effect of displacement bias correction is more apparent. HC alone reduces heading drift but does not correct for translation errors caused by systematic displacement undershoot. As shown in Figure 5, realized gait cycle displacements on sand consistently undershoot the commanded displacement. This causes the naive and HC dead reckoning estimates to compress relative to the intended pattern, even when heading is corrected (Figure 7a). The HC + NN algorithm reduces the dead reckoning drift by using body-frame acceleration profiles to infer command-to-displacement bias for each two-step gait cycle. The result supports the hypothesis that IMU acceleration profiles contain substrate-dependent command execution errors that are not corrected by heading correction alone. The larger improvement on sand is also related to the composition of the training data. Although properties of outdoor sand differ from those of the indoor sand bed, both substrates produce similar gait-substrate interaction effects, such as dactyl sinkage and slip. These effects are represented in the indoor sand training trials, and the CNN bias estimator encounters similar acceleration profile signatures during outdoor deployments.

Trials on sand using CNN displacement bias correction show greater trial-to-trial variability than trials with HC. This variability is explained because the bias estimator operates at the gait cycle level and responds differently to the measured acceleration profile from each individual cycle. Disturbances such as local surface irregularities, variations in dactyl penetration depths, and differences in body motion produce different per-cycle corrections across trials. Despite this increase in variability, HC + NN reduces dead reckoning drift more on sand compared to HC (Figure 8), indicating that body-frame acceleration profiles contain measurable signatures associated with displacement undershoot on deformable terrain.

Several limitations are considered when analyzing the results. First, the current CNN bias estimator is platform- and gait-specific. The alternating tripod gait provides data at fixed cycle intervals during training and evaluation, which might be simpler to analyze than a more irregular planning walk (Chen et al., 2022) or reactive gait (Azayev and Zimmerman, 2020). Different gaits produce different leg contact sequences and acceleration profiles, requiring either gait-specific retraining or a new combined model. To address the effect of gait changes, future work may compare affect of different step periods. There may be trade-offs in which more complex gaits are more efficient in traversing complex terrain but simpler gaits are better for estimating progress. Second, acceleration input is retained only along the commanded direction. Since the dominant error on level sand is slip-induced undershoot along the commanded direction, the acceleration along the commanded direction is used. Lateral and vertical components may contain gait-induced oscillations that are less directly related to planar displacement. On surfaces with various elevation grades, down-slope sliding may introduce lateral and vertical displacement errors, making lateral and vertical acceleration components important for three-dimensional bias correction. Finally, the magnetometer-derived yaw provides an absolute heading reference and limits unbounded drift under normal magnetic conditions. However, the current system does not reject external magnetic disturbances that affect the entire array. Previous studies have demonstrate that Hall effect sensors achieve terrain and object classification (Grezmak et al., 2021; Grezmak and Daltorio, 2024). Such measurements may complement the body mounted IMUs by providing gait-terrain interaction information for terrain-aware displacement bias correction. To mitigate yaw error accumulation during long term missions, periodic heading or position correction from independent sources may eventually be necessary, as the proposed method focuses on following commanded patterns for local searches.

## 5 Conclusion

This work demonstrates that low-cost multi-IMU sensing enables more accurate planar dead reckoning for an encoder-free 18-DoF hexapod platform performing local searches on soft and granular media. Since an encoder-free robot walks imprecisely, commanded kinematics alone does not accurately predict the realized body motion, especially when substrate-dependent sinkage or slip occur. To reduce dead reckoning drift, we combine a low-cost IMU array (Figure 1) with a planar dead reckoning pipeline (Figure 3) that uses magnetometer-aided heading correction and CNN displacement bias correction (Figure 4). The CNN bias estimator is trained using combined indoor foam tiles and sand bed data, which is then applied to outdoor grass and sand without explicit terrain labels or model switching. Outdoor tests show that the proposed correction method reduces dead reckoning drift on both grass (Figure 6) and sand (Figure 7), with overall drift reductions by 41% on grass and 59% on sand relative to the naive dead reckoning baseline (Figure 8).

The results suggest that body-mounted IMU acceleration profiles contain useful motion signatures associated with command-to-displacement bias during encoder-free locomotion. The CNN bias estimator does not explicitly classify terrain or learn a full terramechanics model. Instead, it maps commanded motions and measured acceleration profiles to a platform-specific per-cycle displacement bias estimate. The larger improvement on sand suggests that the estimator is sensitive to how well indoor training substrate captures locomotion error observed during outdoor deployments. The grass results indicated only partial cross-substrate generalization instead of the full potential of the correction method on that terrain, and it is unclear whether the HC + NN method would outperform HC more noticeably if outdoor grass training data is available.

Future work will focus on several directions. First, the training dataset will be expanded to include grass-covered soil and uneven natural terrain, allowing the CNN bias estimator to learn motion signatures associated with local surface depressions. Additional data on bumpy terrain will also help determine if terrain-induced body oscillations are distinguishable from acceleration profiles associated with displacement undershoot. Second, future experiments will compare gait-specific estimators with a combined multi-gait model across different gait patterns and gait cycle durations. Third, synchronized individual IMU measurements will be used to quantify how sensor count and placement affect estimation accuracy. Mitigating magnetic interference will also be studied to ensure long-term mission reliability. Potential approaches include adapting the weight of magnetometer measurements during suspected disturbances and temporarily relying on other heading measurements until normal magnetic condition restores. Finally, the framework will be extended to three-dimensional dead reckoning by evaluating acceleration features that are parallel, orthogonal, and vertical to the direction of motion. A three-dimensional displacement bias estimator will be constructed using ground truth trajectories collected on sloped and uneven terrain. The resulting framework will be evaluated in underwater environments, representing the original use case of the platform.

## Data Availability

The raw data supporting the conclusions of this article will be made available by the authors, without undue reservation.
